# Treatment strategy for spontaneous coronary artery dissection based on anatomical characteristics

**DOI:** 10.1186/s40001-023-00986-y

**Published:** 2023-01-16

**Authors:** Yuanji Ma, Xin Zhong, Jiasheng Yin, Hao Lu, Congcong Pan, Dong Huang, Junbo Ge

**Affiliations:** 1 Department of Cardiology, Zhongshan Hospital, Fudan University, 1609 Xietu Road, Shanghai, 200032 China; 2 Shanghai Institute of Cardiovascular Diseases, 1609 Xietu Road, Shanghai, 200032 China; 3 National Clinical Research Center for Interventional Medicine, 1609 Xietu Road, Shanghai, 200032 China

**Keywords:** Spontaneous coronary artery dissection, Percutaneous coronary intervention, Conservative treatment, Vessel healing

## Abstract

**Objectives:**

To compare the clinical and angiographic characteristics of high-risk and low-risk spontaneous coronary artery dissection (SCAD) patients to determine the optimal treatment strategy.

**Background:**

SCAD is a rare and emerging cause of acute coronary syndrome and sudden cardiac death, especially in young female patients. However, the indication of percutaneous coronary intervention (PCI) in patients with SCAD remains elusive.

**Methods:**

We evaluated the clinical and angiographic characteristics of all SCAD patients admitted to our center from 2012 to 2020. The outcomes of the high-risk and low-risk SCAD patients according to the location of the lesion segment with dissection or intramural hematoma were compared. Further analyses were performed to evaluate the vessel healing or residual dissection in the patients receiving the follow-up angiography.

**Results:**

A total of 81 SCAD patients were enrolled in the present study, in which 38 patients were categorized as high-risk group, defined as involvement of the left main artery or proximal segment of any main coronary artery. PCI was the more common treatment approach in the high-risk group (68.4%), while conservative treatment was more common in the low-risk group (62.8%). The incidence of major adverse cardiac events, defined as cardiac death, myocardial infarction, unstable angina pectoris, severe arrhythmias, or heat failure, within 1 year follow-up was similar between the two groups. 57 patients (70.4%) received the follow-up angiography after 1 year. The high- and low-risk groups had a similar rate of vessel healing among the PCI treatment patients. However, more patients achieved spontaneous healing in the low-risk group than the high-risk group among the conservative treatment patients (86.4% vs. 33.3%, *p* < 0.05).

**Conclusions:**

Conservative management remains the recommended treatment strategy for the low-risk SCAD patients. PCI could be considered in high-risk SCAD patients with favorable clinical outcomes and vessel healing. Characterization of lesion anatomy may be an important indicator for treatment decision.

## Introduction

Spontaneous coronary artery dissection (SCAD) has emerged as an important etiology of acute coronary syndrome (ACS) and sudden cardiac death, especially in the younger patient population, with female predominance [1]. Due to the rarity of the disease, SCAD is often underdiagnosed or misdiagnosed. The accurate definition of SCAD is an epicardial coronary artery dissection, with exclusion of atherosclerotic, iatrogenic, or traumatic causes. The main mechanism of SCAD is coronary artery obstruction caused by formation of an intramural hematoma (IMH) or intimal disruption rather than atherosclerotic plaque rupture or intraluminal thrombosis [2, 3]. However, the true etiology of SCAD may be multifactorial, including gender, hormone secretion, genetic predisposition, environmental, emotional triggers, or underlying vascular pathology, such as fibromuscular dysplasia [4, 5].

In view of the low diagnostic rate, the true prevalence of SCAD remains unclear. Unseasoned clinicians may be unfamiliar with the coronary angiographic (CAG) presentation of SCAD and can often be misdiagnosed as atherosclerosis-related dissection in patients with ACS. Aside from iatrogenic, traumatic and atherosclerotic dissection, about 1–4% of ACS are caused by SCAD [6, 7]. Recent studies have shown that the left anterior descending (LAD) artery is the most commonly affected coronary artery in SCAD, in which the middle and distal segments are the most common lesion site [8, 9].

In contrast to atherosclerotic ACS, the indication of percutaneous coronary intervention (PCI) in patients with SCAD remains elusive. Previous studies have shown that PCI in SCAD is associated with a higher risk of procedural complications, including iatrogenic dissection, acute vascular occlusion, hematoma extension or unplanned stents implantation. Therefore, the American Heart Association (AHA) and European Society of Cardiology (ESC) Statements on SCAD both recommend conservative management over PCI in most SCAD cases [10, 11]. However, in SCAD patients presenting with high-risk coronary anatomy or compelling clinical scenario, the revascularization strategy, including coronary artery bypass grafting (CABG) or PCI should be considered [11]. However, the definition of high-risk anatomical characteristics and the relevant treatment strategy remain debatable.

The present study retrospectively analyzed the clinical and angiographic data of SCAD patients to investigate whether specific high-risk anatomical clusters can be identified, which may help the individually tailored treatment strategies in patients who experienced SCAD.

## Methods

### Patient population

Patients with SCAD were recruited between 2012 and 2020 from the electronic medical database of Zhongshan Hospital. The patients diagnosed with coronary artery dissection through CAG were initially screened for eligibility. Patients with atherosclerotic, traumatic or iatrogenic dissection were collectively excluded. Angiographic and intravascular imaging data were reviewed by two independent, experienced interventional cardiologists to identify the anatomical characteristics of the affected vessel and segment. Any dispute or disagreements were settled with an open discussion. Eligible participants were contacted via e-mail or telephone and were asked to provide digital informed consent to examine their hospital records.

### Angiographic findings

The culprit lesion was classified based on the Saw angiographic SCAD classification. Type 1 refers to classical appearance of multiple radiolucent lumens or arterial wall contrast staining, Type 2 refers to the presence of diffuse stenosis that can vary in severity and length, Type 3 presents as focal or tubular stenosis, that mimics atherosclerosis. Intracoronary imaging is helpful in confirming a true or false lumen and intramural hematoma, aiding with assessment of vessel dimensions [11]. The high- or low-risk SCAD were categorized by the location of the lesion segment with dissection or intramural hematoma. Involvement of the left main artery or proximal segment of any main coronary artery were considered as high-risk SCAD. Involvement of the side branch or the middle and distal segments of the main coronary artery were defined as low-risk SCAD. Other coronary imaging details, including initial thrombolysis in myocardial infarction (TIMI) flow grade, lesion length, percent of stenosis, and presence of atherosclerosis or thrombus, were also collected.

### Treatment methods

The method and device for PCI, including stent implantation and/or balloon angioplasty, were recorded. The medical records of the patients received conservative treatment or CABG were also analyzed. All other evidence-based therapies recommended by the guidelines, including statin, beta-blockers, and antiplatelet therapy, were recorded during index hospitalization and follow-up.

### Endpoints

Major adverse cardiac events (MACE) were defined as cardiac death, myocardial infarction, unstable angina pectoris, severe arrhythmias, or heat failure documented within 1 year follow-up. Further analyses were performed on patients who received follow-up CAG to evaluate vessel healing or residual dissection. The residual dissection in the patients receiving the stenting is defined as stent malposition, in-stent restenosis, stent thrombosis, persistent or extension intramural hematoma, or unplanned target vessel revascularization.

### Statistical analysis

All statistical analyses were performed with SPSS 23 (IBM Corporation, USA). Categorical variables were presented as frequency (%), while continuous variables as mean ± standard deviation. Categorical variables were compared with Pearson’s correlation or the Chi-square test, while continuous variables were compared using the independent Student’s *t* test. A Kaplan–Meier analysis and log-rank test were used to display the incidence of MACE during 1 year follow-up. All statistical analyses were two-sided with a given *p* value of < 0.05 considered as statistically significant.

## Results

### Patient characteristics

**Fig. 1:**
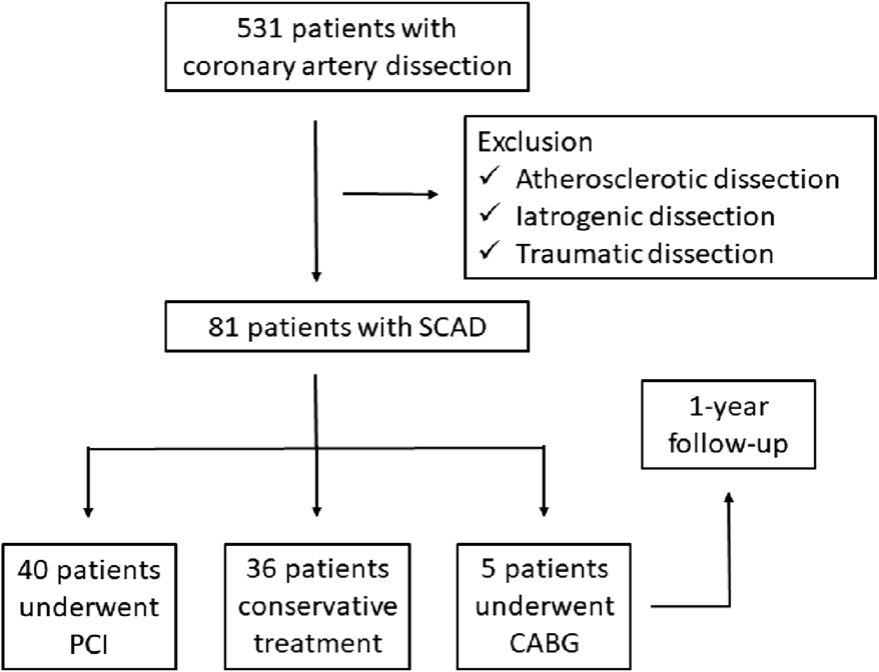
Flow chart of study enrollment

A total of 531 coronary artery dissection patients were screened for inclusion, and 450 patients were subsequently excluded after careful review of the medical history and coronary angiographic or intravascular imaging data. A total of 81 patients diagnosed with SCAD were included in the present study (Fig. 1). Over half of the patients (67.9%) were female, with an average age of 56.8y. Few of the patients have traditional coronary risk factors. However, 95.1% of the patients presented as ACS upon admission, in which ST-elevation myocardial infarction (STEMI) was 23.5%. Only 4.9% of the patients presented as stable angina (SA). Detailed baseline characteristics are summarized in Table 1.

**Table 1:** Baseline characteristics and comparison between high-risk and low-risk SCAD

	Global population (*n* = 81)	High-risk (*n* = 38)	Low-risk (*n* = 43)	*p *value
Clinical data				
Age	56.83 ± 12.77	54.87 ± 11.30	58.56 ± 13.85	0.196
Gender				
Female	55 (67.9)	26 (68.4)	29 (67.4)	0.925
Male	26 (32.1)	12 (31.6)	14 (32.6)	
Coronary risk factor				
Hypertension	40 (49.4)	16 (42.1)	24 (55.8)	0.218
Hyperlipidemia	22 (27.2)	9 (23.7)	7 (16.3)	0.404
Diabetes mellitus	16 (19.8)	14 (36.8)	8 (18.6)	0.066
Smoking	18 (22.2)	8 (21.1)	10 (23.3)	0.768
Clinical presentation				
NSTEMI	11 (13.6)	2 (5.3)	9 (20.9)	0.237
STEMI	19 (23.5)	10 (26.3)	9 (20.9)	
UA	47 (58.0)	24 (63.2)	23 (53.5)	
SA	4 (4.9)	2 (5.3)	2 (4.7)	
Laboratory data				
cTnT (ng/ml)	0.18 ± 0.50	0.15 ± 0.51	0.20 ± 0.49	0.596
CK-MB (U/L)	17.65 ± 14.69	19.43 ± 19.94	16.12 ± 7.83	0.331
NT-proBNP (pg/ml)	499.15 ± 639.84	369.77 ± 419.90	610.47 ± 769.08	0.094
D-dimer (mg/L)	0.57 ± 1.00	0.33 ± 0.33	0.80 ± 1.33	0.054
EF (%)	59.58 ± 8.77	59.62 ± 8.70	59.54 ± 8.96	0.967
CAG findings				
Affected vessel				
LAD	26 (32.1)	9 (23.7)	17 (39.5)	0.021
LCX	8 (9.9)	1 (2.6)	7 (16.3)	
RCA	45 (55.6)	26 (68.4)	19 (44.2)	
LM	2 (2.5)	2 (5.3)	0 (0)	
Affected segment				
Proximal	38 (46.9)		/	/
Middle	30 (37.0)	/	/	/
Distal	13 (16.1)	/	/	/
Saw classification				
Type 1	47 (58.0)	24 (63.2)	23 (53.5)	0.240
Type 2	30 (37.1)	11 (29.0)	19 (44.2)	
Type 3	4 (4.9)	3 (7.9)	1 (2.3)	
Coronary analysis				
Stenosis (%)	68.46 ± 24.24	71.90 ± 23.94	65.42 ± 24.38	0.232
Length (mm)	40.12 ± 24.33	34.08 ± 23.51	45.47 ± 24.05	0.035
Initial TIMI flow				
0	9 (11.1)	4 (10.5)	5 (11.6)	0.502
1	4 (4.9)	2 (5.3)	2 (4.7)	
2	2 (2.5)	2 (5.3)	0 (0)	
3	66 (81.5)	30 (78.9)	36 (83.7)	
Atherosclerosis	35 (43.2)	17 (44.7)	18 (41.9)	0.794
Thrombus	6 (7.4)	3 (7.9)	3 (7.0)	0.875
Treatment method				
PCI	40 (49.4)	26 (68.4)	14 (32.5)	0.002
Conservative treatment	36 (44.4)	9 (23.7)	27 (62.8)	
CABG	5 (6.2)	3 (7.9)	2 (4.7)	
Number of stents	1.89 ± 1.11	1.58 ± 1.02	2.55 ± 1.04	0.014
Follow-up				
1 year MACE	10 (12.3)	6 (15.8)	4 (9.3)	0.376
UA	6 (7.4)	3 (7.9)	3 (7.0)	
Heart failure	3 (3.7)	2 (5.3)	1 (2.3)	
Cardiac death	1 (1.2)	1 (2.6)	0 (0)	

Values are mean ± SD or *n* (%)

*SCAD* spontaneous coronary artery dissection, *CAG* coronary angiography, *NSTEMI* non-ST segment elevation myocardial infarction, *STEMI* ST segment elevation myocardial infarction, *UA* unstable angina, *SA* stable angina, *cTnT* cardiac troponin T, *CK-MB* creatine kinase MB isoenzyme, *NT-proBNP* N-terminal pro-B-type natriuretic peptide, *EF* ejection fraction, *LAD* left anterior descending artery, *LCX* left circumflex artery, *RCA* right coronary artery, *LM* left main artery, *PCI* percutaneous coronary intervention, *CABG* coronary artery bypass grafting

### Angiographic and procedural characteristics

**Fig. 2:**
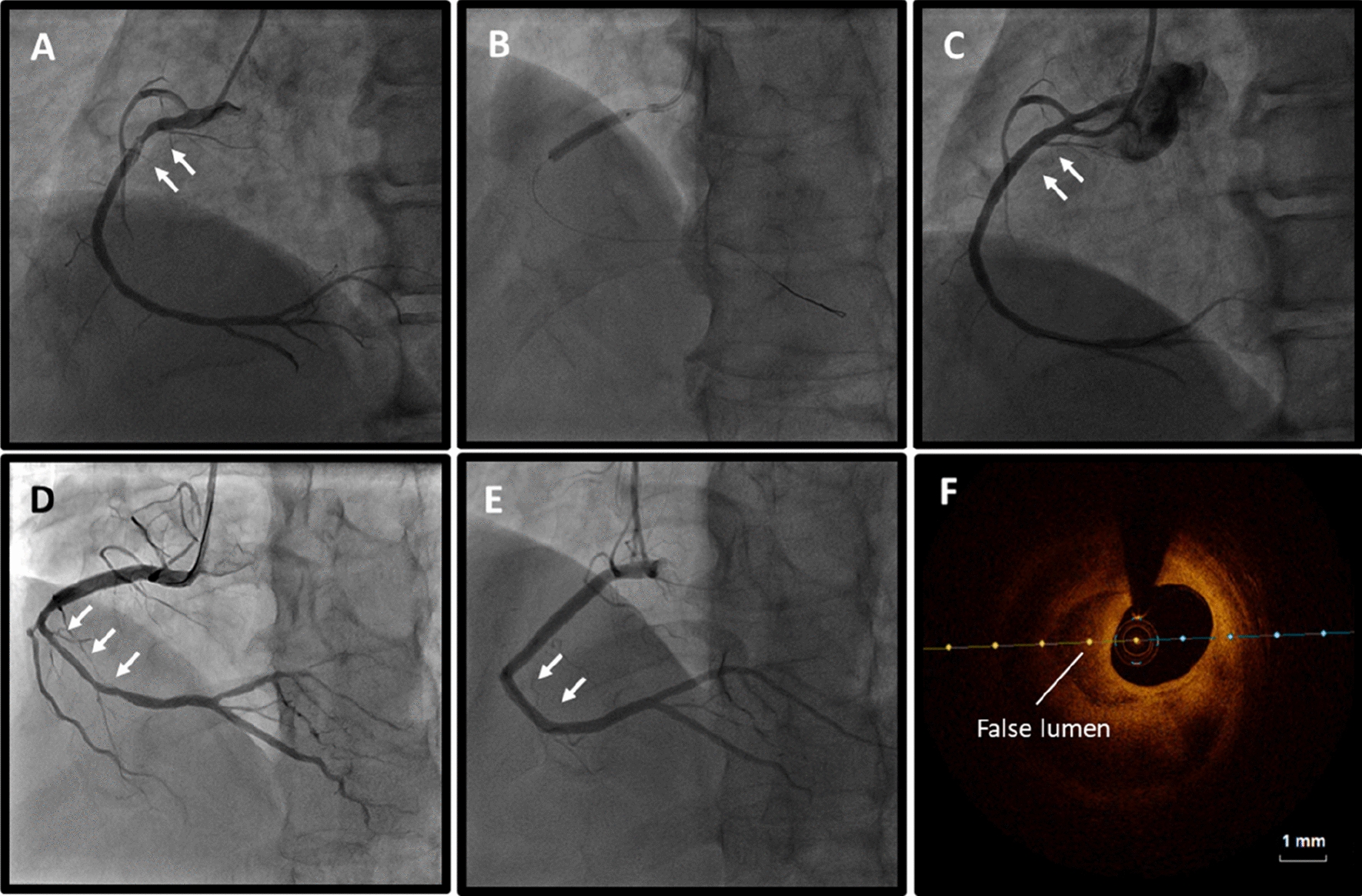
Coronary angiography in patients with SCAD. **A** SCAD patients presenting with high-risk coronary anatomy (proximal RCA). **B** Implantation of drug-eluting stent. **C** Repeat angiography after initial PCI. **D **SCAD patients presenting with low-risk coronary anatomy (middle-distal RCA). **E** Repeat angiography 12 month after conservative treatment. **F** OCT images visualized the true lumen and the false lumen of SCAD. The white arrows indicate SCAD in RCA. *SCAD* spontaneous coronary artery dissection, *RCA* right coronary artery, *PCI* percutaneous coronary intervention, *OCT* optical coherence tomography

According to the Saw angiographic SCAD classification, the percentage of type 1, 2 and 3 were 58.0%, 37.1% and 4.9%, respectively. Majority of SCAD were found in right coronary artery (RCA, 55.6%), followed by LAD (32.1%), left circumflex (LCX, 9.9%) and left main (LM, 2.5%) artery. Affected segment was determined via CAG examination and divided into proximal (46.9%), middle (37.0%) and distal (16.1%). Involvement of the LM or proximal segment of any main coronary artery were found in approximately half of the patients (46.9%), which was classified as high-risk SCAD group. Most of patients (81.5%) had the initial TIMI grade 3 flow. About half of the patients (49.4%) patients received PCI and 44.4% of the patients were managed conservatively. The incidence of MACE after 1 year follow-up was 12.3%. Figure 2 shows the representative cases of SCAD receiving PCI or conservative management.

### The high-risk and low-risk SCAD

**Fig. 3:**
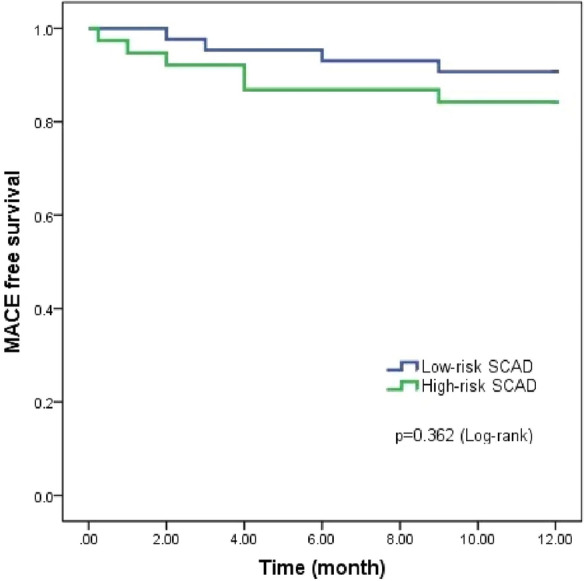
Incidence of MACE for patients with high-risk SCAD (green line) versus low-risk SCAD (blue line). Abbreviations as in Fig. 2

No significant differences were observed between the baseline clinical characteristics of the high-risk and low-risk SCAD groups (Table 1). High-risk group had a higher rate of RCA involvement than the low-risk group (68.4% vs. 44.2%, *p* < 0.05), while the culprit vessel in the low-risk group was more frequently located in LAD and LCX (39.5% vs. 23.7% and 16.3% vs. 2.6%) (*p* < 0.05). The length of the dissection was shorter in the high-risk group than the low-risk group (34.1 vs. 45.5 mm, *p* < 0.05). More patients in the high-risk group received PCI (68.4% vs. 32.5%, *p* < 0.01), while most patients in the low-risk SCAD group received conservative management (62.8% vs. 23.7%, *p* < 0.01) (Table 1). However, the incidence of MACE after 1 year follow-up was comparable between the two groups (Fig. 3).

Among 40 patients who received PCI, 26 patients were categorized as high-risk group (Table 2). Cardiac troponin T (cTnT) level was higher and more stents were placed in the low-risk group (0.38 vs. 0.03 ng/ml, 2.55 vs. 1.58, *p* < 0.05, Table 2). Among 36 patients with conservative management, 9 patients were categorized as high-risk group (Table 3). The levels of cTnT and creatine kinase MB isoenzyme (CK-MB) were higher in the high-risk group (0.52 vs. 0.12 ng/ml, 33 vs. 16 U/L, *p* < 0.05, Table 3). Involvement of the LAD was more common in the low-risk group (51.9% vs. 22.2%, *p* < 0.05), while involvement of RCA was more common in the high-risk group (77.7% vs. 29.6%, *p* < 0.05, Table 3).

**Table 2:** Subgroup analysis of PCI group

	PCI treatment (*n* = 40)	High-risk (*n* = 26)	Low-risk (*n* = 14)	*p *value
Clinical data				
Age	56.68 ± 11.60	57.19 ± 11.74	55.71 ± 11.72	0.706
Gender				
Female	30 (75.0)	19 (73.1)	11 (78.6)	0.702
Male	10 (25.0)	7 (26.9)	3 (21.4)	
Coronary risk factor				
Hypertension	19 (47.5)	11 (42.3)	8 (57.1)	0.370
Hyperlipidemia	10 (25.0)	7 (6.9)	3 (21.4)	0.702
Diabetes mellitus	13 (32.5)	11 (42.3)	2 (14.3)	0.071
Smoking	7 (17.5)	5 (19.2)	2 (14.3)	0.695
Clinical presentation				
NSTEMI	2 (5.0)	1 (3.8)	1 (7.1)	0.538
STEMI	10 (25.0)	6 (23.1)	4 (28.6)	
UA	25 (62.5)	18 (69.2)	7 (50.0)	
SA	3 (7.5)	1 (3.8)	2 (14.3)	
Laboratory data				
cTnT (ng/ml)	0.15 ± 0.50	0.03 ± 0.05	0.38 ± 0.80	0.031
CK-MB (U/L)	15 ± 6.44	15.04 ± 3.96	16.31 ± 9.54	0.579
NT-proBNP (pg/ml)	429.40 ± 630.90	314.58 ± 433.47	634.42 ± 863.69	0.131
D-dimer (mg/L)	0.35 ± 0.33	0.32 ± 0.33	0.42 ± 0.34	0.447
EF (%)	62.19 ± 7.23	62.16 ± 7.17	62.25 ± 7.68	0.972
Angiographic findings				
Culprit vessel				
LAD	9 (22.5)	7 (26.9)	2 (14.3)	0.364
LCX	3 (7.5)	1 (3.8)	2 (14.3)	
RCA	26 (65.0)	16 (61.5)	10 (71.4)	
LM	2 (5.0)	2 (7.7)	0 (0)	
Affected segment				
Proximal	24 (60.0)	/	/	/
Middle	9 (22.5)	/	/	/
Distal	5 (12.5)	/	/	/
Saw classification				
Type 1	24 (60.0)	17 (65.4)	7 (50.0)	0.408
Type 2	15 (37.5)	8 (30.8)	7 (50.0)	
Type 3	1 (2.5)	1 (3.8)	0 (0)	
Coronary analysis				
Stenosis (%)	78.50 ± 20.60	77.77 ± 20.81	79.86 ± 20.89	0.764
Length (mm)	38.50 ± 22.31	34.62 ± 21.16	45.71 ± 23.36	0.135
Initial TIMI flow				
0	6 (15.0)	3 (11.5)	3 (21.4)	0.539
1	4 (10.0)	2 (7.7)	2 (14.3)	
2	2 (5.0)	2 (7.7)	0 (0)	
3	28 (70.0)	19 (73.1)	9 (64.3)	
Atherosclerosis	18 (45.0)	10 (38.5)	8 (57.1)	0.257
Thrombus	4 (10.0)	2 (7.7)	2 (14.3)	0.507
Treatment method				
Number of stents	1.89 ± 1.11	1.58 ± 1.02	2.55 ± 1.04	0.014
Follow-up				
1 year MACE	3 (7.5)	3 (11.5)	0 (0)	0.186
UA	2 (5)	2 (7.7)	0 (0)	
Heart failure	0 (0)	0 (0)	0 (0)	
Cardiac death	1 (2.5)	1 (3.8)	0 (0)	

Values are mean ± SD or *n* (%)

Abbreviations as in Table 1

**Table 3:** Subgroup analysis of conservative treatment group

	Conservative treatment (*n* = 36)	High-risk (*n* = 9)	Low-risk (*n* = 27)	*p *value
Clinical data				
Age	56.28 ± 13.98	48.11 ± 8.98	59.00 ± 14.41	0.041
Gender				
Female	20 (55.6)	4 (44.4)	16 (59.3)	0.439
Male	16 (44.4)	5 (55.6)	11 (40.7)	
Coronary risk factor				
Hypertension	19 (52.8)	4 (44.4)	15 (55.5)	0.563
Hyperlipidemia	6 (16.7)	2 (22.2)	4 (14.8)	0.606
Diabetes mellitus	7 (19.4)	2 (22.2)	5 (18.5)	0.808
Smoking	11 (30.6)	3 (33.3)	8 (29.6)	0.886
Clinical presentation				
NSTEMI	8 (22.2)	1 (11.1)	7 (25.9)	0.213
STEMI	8 (22.2)	3 (33.3)	5 (18.5)	
UA	19 (52.8)	4 (44.4)	15 (55.5)	
SA	1 (2.8)	1 (11.1)	0 (0)	
Laboratory data				
cTnT (ng/ml)	0.22 ± 0.53	0.52 ± 0.99	0.12 ± 0.20	0.050
CK-MB (U/L)	20.42 ± 20.11	33.11 ± 36.88	16.19 ± 7.19	0.027
NT-proBNP (pg/ml)	565.69 ± 646.55	515.02 ± 326.75	582.59 ± 727.12	0.790
D-dimer (mg/L)	0.80 ± 1.39	0.25 ± 0.14	0.96 ± 1.55	0.238
EF (%)	57 ± 9.75	53.78 ± 9.67	58.16 ± 9.71	0.254
Angiographic findings				
Culprit vessel				
LAD	16 (44.4)	2 (22.2)	14 (51.9)	0.034
LCX	5 (13.9)	0 (0)	5 (18.5)	
RCA	15 (41.7)	7 (77.7)	8 (29.6)	
LM	0 (0)	0 (0)	0 (0)	
Affected segment				
Proximal	9 (25.0)	/	/	/
Middle	19 (52.8)	/	/	/
Distal	8 (22.2)	/	/	/
Saw classification				
Type 1	20 (55.6)	5 (55.5)	15 (55.5)	0.181
Type 2	13 (36.1)	2 (22.2)	11 (40.7)	
Type 3	3 (8.3)	2 (22.2)	1 (3.7)	
Coronary analysis				
Stenosis (%)	58.53 ± 23.81	54.44 ± 27.09	57.22 ± 23.14	0.767
Length (mm)	43.89 ± 27.15	37.22 ± 32.32	46.11 ± 25.51	0.403
Initial TIMI flow				
0	2 (5.6)	0 (0)	2 (7.4)	0.401
1	0 (0)	0 (0)	0 (0)	
2	0 (0)	0 (0)	0 (0)	
3	34 (94.4)	9 (100)	25 (92.6)	
Atherosclerosis	13 (36.1)	5 (55.6)	8 (29.6)	0.161
Thrombus	2 (5.6)	1 (11.1)	1 (3.7)	0.401
Follow-up				
1 year MACE	5 (13.9)	2 (22.2)	3 (11.1)	0.404
UA	3 (8.3)	0 (0)	3 (11.1)	
Heart failure	2 (5.6)	2 (22.2)	0 (0)	
Cardiac death	0 (0)	0 (0)	0 (0)	

Values are mean ± SD or *n* (%)

Abbreviations as in Table 1

### Vessel healing analysis

Repeat CAG were performed in 57 patients (70.4%), in which 29 patients received PCI and 28 patients received conservative treatment (Table 4). Among the PCI treatment patients, the high- and low-risk groups had a similar rate of vessel healing. However, more patients achieved spontaneous vessel healing in the low-risk group than the high-risk group among the conservative treatment patients (86.4% vs. 33.3%, *p* < 0.05). It means that a higher rate of residual dissection was observed in the high-risk group than the low-risk group among the conservative treatment patients (66.7% vs. 13.6%, *p* < 0.01, Table 4).

**Table 4:** Repeat CAG results of different treatment methods

	Global population (*n* = 57)	High-risk (*n* = 20)	Low-risk (*n* = 9)	*p *value
Repeat CAG				
SCAD healing	40 (70.2)	16 (61.5)	24 (77.4)	0.449
Residual dissection	17 (29.8)	10 (38.5)	7 (22.6)	

	PCI treatment (*n* = 29)	High-risk (*n* = 20)	Low-risk (*n* = 9)	*p*-value
Repeat CAG				
SCAD healing	19 (65.5)	14 (70.0)	5 (55.6)	0.449
Residual dissection	10 (34.5)	6 (30.0)	4 (44.4)	

	Conservative Treatment (*n* = 28)	High-risk (*n* = 6)	Low-risk (*n* = 22)	*p-*value
Repeat CAG				
Spontaneous healing	21 (75.0)	2 (33.3)	19 (86.4)	0.008
Residual dissection	7 (25.0)	4 (66.7)	3 (13.6)	

Values are n (%)

Abbreviations as in Table 1

## Discussion

The present study is the first to compare the clinical characteristics and treatment efficacy of high-risk and low-risk SCAD based on lesion anatomy. High-risk SCAD is defined as dissection involving the LM and the proximal segment of any main coronary artery with a slight modification from previous definition [11]. Conservative management remains the recommended treatment strategy for the low-risk SCAD patients with a high rate of spontaneous vessel healing. PCI in the high-risk SCAD patients could achieve the favorable clinical outcomes and relatively similar vessel healing with the conservative treatment.

Although SCAD belongs to the broad spectrum of coronary heart disease, its treatment strategy is different from traditional CAD [2, 3]. Based on current recommendations, most of the clinically stable patients should consider the conservative management. Only those with ongoing ischemia or hemodynamic instability should consider urgent PCI or CABG [11]. However, emergency CABG is only feasible in certain medical facilities, while emergency PCI has a more widespread coverage. About 5–10% of patients who receive conservative treatment experience early recurrence of MI, which is often related to extension of dissection within the first 7 days after an acute ACS episode [12]. Previous study [13] showed that most SCAD patients undergoing PCI were high risk at presentation, including STEMI, cardiac arrest, TIMI 0/1 flow or proximal dissections. Conservative management in these patients may not be appropriate and can incur greater risks. Several studies have proven the value of PCI in SCAD, especially in high-risk coronary lesions, with promising outcome. Patients with STEMI–SCAD had more favorable prognoses in revascularization management modalities than those who with atherosclerosis-related STEMI [13–16]. In the present study, the high-risk and low-risk patients had the similar clinical characteristics, including ACS presentation and female predominance. The incidence of 1 year MACE was comparable between the two groups. Due to the retrospective nature of the present study, the appropriate treatment method was determined by the operator. Interestingly, PCI was employed in majority of the high-risk cases, while most of the low-risk cases received conservative management. High-risk patients in the present study underwent PCI treatment for SCAD with low complication rates and similar outcome with the low-risk patients. Several important technical factors, including careful manipulation of guiding catheter and wire, direct stenting without balloon predilation, long stents to cover the proximal and distal ends of the hematoma by 5–10 mm, and guidance with intravascular ultrasound (IVUS), could limit the hematoma propagation-related complications during PCI management of SCAD.

A small portion of the low-risk SCAD patients received PCI in the present study. In SCAD patients who received PCI, cTnT (ng/ml) values were higher in the low-risk group than the high-risk group, which was associated with a higher rate of baseline TIMI 0/1 flow and urgent decision of PCI. This may be associated with a more severe clinical feature, which prompted the clinical decision of PCI. In comparison with the conservative treatment group, cTnT (ng/ml) and CK-MB (U/L) values were higher in the high-risk group (*n* = 9). Regardless, all 9 patients had a TIMI 3 flow and, therefore, received conservative management and close follow-up. Previous studies have indicated angiographic “healing” of SCAD lesions is plausible in majority of patients after a conservatively managed index episode [8, 9]. According to the follow-up CAG results in our study, majority of the patients in the high-risk group receiving PCI achieved vessel healing. Conservative management was also associated with a significantly higher rate of spontaneous vessel healing in low-risk patients. Therefore, we propose that conservative management is the acceptable treatment approach but should be carefully selected for patients with low-risk SCAD anatomy and stable clinical features. In patients presenting with high-risk SCAD anatomy or compelling clinical scenario, emergency revascularization can be achieved with PCI.

Compared with low-risk lesions, patients with proximal lesions cover a larger myocardial blood supply area. Recent study suggested SCAD involving the proximal coronary arteries was associated with a reduction in post-infarct ejection fraction [17]. This may be associated with a more severe clinical feature, which prompted the clinical decision of PCI. In the present study, no significant difference in Saw classification was noted between high-risk and low-risk groups. Although Saw classification has a high diagnostic value, it cannot provide the reference for treatment decisions in patients with SCAD [18–21].

There are several limitations in the present study. First, this present study summarizes the experience of a single center, with a rather small sample size. Second, the retrospective nature of the study may incur selection bias especially in treatment methods. Third, this study lacks long-term follow-up data for SCAD patients with different interventions. Future prospective randomized control trial including multi-center data is warranted to validate the present study results.

The present study proposed a new classification method based on lesion segment and anatomical characteristics of SCAD, providing a simple and convenient alternative for interventional cardiologists to quickly formulate a suitable treatment strategy for patients in emergency situations. PCI may be chosen in patients with SCAD involvement of the LM or proximal segment of any main coronary artery. While conservative treatment should be reserved for low-risk SCAD patients with the involvement of the side branch or the middle and distal segments of the main coronary artery.

## Conclusion

This study provides insight to the treatment strategy of SCAD patient based on lesion anatomy and affected segment. Conservative management remains the recommended treatment strategy for the low-risk SCAD patients. PCI could be considered in high-risk SCAD patients with favorable clinical outcomes and vessel healing.

## Data Availability

Original data could be made available by formal acquisition to the corresponding author.

## Abbreviations

SCAD: Spontaneous coronary artery dissection
PCI: Percutaneous coronary intervention
MACE: Major adverse cardiac events
ACS: Acute coronary syndrome
IMH: Intramural hematoma
CAG: Coronary angiography
TIMI: Initial thrombolysis in myocardial infarction
LAD: Left anterior descending artery
LCX: Left circumflex artery
RCA: Right coronary artery
LM: Left main artery
CABG: Coronary artery bypass grafting
STEMI: ST segment elevation myocardial infarction
IVUS: Intravascular ultrasound
cTnT: Cardiac troponin T
CK-MB: Creatine kinase MB isoenzyme
